# Gene Expression Profile in the Long-Living Lotus: Insights into the Heat Stress Response Mechanism

**DOI:** 10.1371/journal.pone.0152540

**Published:** 2016-03-28

**Authors:** Xiaojing Liu, Fengfeng Du, Naiwei Li, Yajun Chang, Dongrui Yao

**Affiliations:** Institute of Botany, Jiangsu Province and Chinese Academy of Sciences, Nanjing 210014, China; Youngstown State University, UNITED STATES

## Abstract

Lotus (*Nelumbo Adans*) is an aquatic perennial plant that flourished during the middle Albian stage. In this study, we characterized the digital gene expression signatures for China Antique lotus under conditions of heat shock stress. Using RNA-seq technology, we sequenced four libraries, specifically, two biological replicates for control plant samples and two for heat stress samples. As a result, 6,528,866 to 8,771,183 clean reads were mapped to the reference genome, accounting for 92–96% total clean reads. A total of 396 significantly altered genes were detected across the genome, among which 315 were upregulated and 81 were downregulated by heat shock stress. Gene ontology (GO) enrichment of differentially expressed genes revealed protein folding, cell morphogenesis and cellular component morphogenesis as the top three functional terms under heat shock stress. Kyoto Encyclopedia of Genes and Genomes (KEGG) analysis led to the identification of protein processing in endoplasmic reticulum, plant-pathogen interactions, spliceosome, endocytosis, and protein export as significantly enriched pathways. Among the upregulated genes, small heat shock proteins (sHsps) and genes related to cell morphogenesis were particularly abundant under heat stress. Data from the current study provide valuable clues that may help elucidate the molecular events underlying heat stress response in China Antique lotus.

## Introduction

Changes in temperature especially heat shock will inevitably affect plant performance, the moisture distribution and biomass of plants, in particular, aquatic plants. More than 200 million years ago, extremely high temperatures drove most Early Triassic plants and animals away from the equator, and probably constituted the major cause of the end-Permian mass extinction [1]. Heat stress is currently considered the major abiotic stress in many areas worldwide. More focus on the heat tolerance of plants is therefore essential. Elucidation of the relationship between excessive temperatures and cellular response is an important step for optimization of thermotolerance in aquatic plants.

Lotus (*Nelumbo Adans*) is a family of aquatic perennial plants that flourished during the middle Albian period [2]. Areas in north-eastern China provided an important refuge for survival of the lotus species during the Quaternary glaciation era [3]. Only two remaining species have survived from the late Cretaceous, specifically, *N*. *nucifera* Gaertn. and *N*. *lutea* Wild [4]. *N*. *nucifera* is mainly distributed and cultivated in Asia and north Oceania while *N*. *lutea* is native to North America. In Asia, the lotus has been cultivated as food for over 7,000 years, and grows in a wide range of climatic zones in China from longitudes of E86° to 133° and latitudes of N18° to 48° [5].

One of the oldest surviving flora, the lotus is a land plant that has adapted to aquatic environments. Unlike the well-characterized model plants, lotus is considered the ancestor of eudicots, and lies outside of the core eudicots [6]. Significant progress has been made in identifying heat-regulated genes and the related pathways in *Arabidopsis*, rice, maize and wheat [7]. However, limited information on the genes and pathways activated in lotus experiencing heat stress conditions is currently available. The lotus seed is extremely tolerant to heat stress, with 13.5% seeds germinating after treatment at 100°C for 24 h, which is destructive to maize seeds [8]. In addition, embryo axes and cotyledons of lotus show heat-hardiness [9]. However, the pathways contributing to the heat response mechanism in long-living lotus remain to be clarified.

Here, we analyzed the gene expression profile of China Antique lotus under conditions of 37°C heat treatment using RNA-seq. Numerous differentially and specifically expressed transcripts of heat-regulated genes were identified. Expression patterns of candidate genes were further validated using quantitative real-time PCR (qRT-PCR). Our results should aid in elucidating the molecular events underlying the heat stress response in China Antique lotus and provide valuable resources for genetic studies on abiotic stress in lotus.

## Materials and Methods

### Sampling

The seed of ‘China Antique’ lotus (over 1000 years old) was initially identified in northeastern China. Vegetatively propagated lotus root was donated by Nanjing Yileen Flower Company. Plants were cultivated in a greenhouse for one month in the Institute of Botany, Jiangsu province and Chinese academy of Sciences. Lotus plants were used as experimental materials. Control plants were cultivated in a growth chamber under continuous light (160–180 μm sec^-1^m^-2^) at 22°C. For heat shock treatment, plants were transferred to 37°C for 1 h under the same light source. Fresh samples of young leaves above water were collected and frozen in liquid nitrogen for further analysis.

### RNA extraction and library preparation for RNA-seq

Total RNA was isolated using TRIzol reagent (Invitrogen, Carlsbad, CA). A total of 3 μg RNA per sample was used as input material. Sequencing libraries were generated using the NEBNext^®^ Ultra^™^ RNA Library Prep Kit for Illumina^®^ (NEB, USA) following the manufacturer’s recommendations. Briefly, mRNA was purified from total RNA using poly-T oligo-attached magnetic beads. Fragmentation was performed using divalent cations under elevated temperature. First-strand cDNA was synthesized with random hexamer primer and M-MuLV reverse transcriptase. Second-strand cDNA synthesis was subsequently performed using DNA Polymerase I and RNase H. Remaining overhangs were converted into blunt ends via exonuclease/polymerase activities. After adenylation of 3’ ends of DNA fragments, NEBNext adaptor with a hairpin loop structure was ligated to prepare for hybridization. To preferentially select cDNA fragments 150–200 bp in length, library fragments were purified with the AMPure XP system (Beckman Coulter, Beverly, USA). Next, 3 μl USER Enzyme (NEB, USA) was incubated with size-selected, adaptor-ligated cDNA at 37°C for 15 min, followed by 5 min at 95°C before PCR. Amplification was performed with Phusion High-Fidelity DNA polymerase, universal PCR primers and index (X) primer. PCR products were subsequently purified (AMPure XP system), and the library quality assessed on the Agilent Bioanalyzer 2100 system.

The clustering of the index-coded samples was performed on a cBot Cluster generation system using TruSeq PE Cluster Kit v3-cBot-HS (Illumina) according to the manufacturer’s instructions. Following cluster generation, library preparations were sequenced on an Illumina 2500 platform and 50 bp single-end reads generated. The raw data are available in NCBI Sequence Read Archive (SRA, http://www.ncbi.nlm.nih.gov/Traces/sra) with accession number SRP070871.

### Analysis and mapping of RNA-seq reads

Raw data in the Fastq format were initially processed through in-house perl scripts. In this step, clean reads were obtained by removing reads containing adapters, empty reads or reads with unknown sequences ‘N’ from raw data. Simultaneously, Q20 and GC contents were calculated from clean data. All downstream analyses were based on high-quality, clean data. Reference genome and gene model annotation files were downloaded directly from the genome website (http://www.ncbi.nlm.nih.gov/genome/genomes/14095). An index of the reference genome was built using Bowtie v2.0.6, and single-end clean reads aligned to the reference genome using TopHat v2.0.9.

### Differential expression analysis

HTSeq v0.5.4p3 was employed to count read numbers mapped to each gene. Reads per Kilobase per Million mapped Reads (RPKM) of individual genes were calculated based on gene length and read counts mapped to each gene. Differential expression analysis was performed using the DESeq R package (1.10.1). The resulting *P*-values were adjusted using the Benjamini and Hochberg’s approach for controlling the false discovery rate. Genes with adjusted *P*-values <0.05 determined using DESeq were assigned as ‘differentially expressed’.

### GO and KEGG enrichment analysis of differentially expressed genes

Gene Ontology (GO) enrichment analysis of differentially expressed genes was implemented using the GOseq R package, correcting for gene length bias [10]. GO terms with corrected *P* values less than 0.05 were considered significantly enriched by differentially expressed genes. For KEGG pathways analysis, we used KOBAS software to determine the statistical enrichment of differentially expressed genes [11–12].

### Quantitative real-time PCR analysis

Quantitative real-time PCR analysis was used to validate gene expression patterns. Under heat stress treatment (37°C), fresh samples of stems and leaves above water were collected at various time-points (0, 0.5, 1, 3, 6 h). Total RNA (1 μg) was used to synthesize cDNA with random primer pd (N)9. The genes and primer pairs for real-time PCR analysis are listed in Table A in S1 File. QRT-PCR was performed in a 15-ul mixture containing SYBR Premix Ex Taq^™^ II (Clontech). PCR was performed as follows: initial denaturation for 30 s at 95°C, followed by 40 cycles of 15 s at 95°C, 15 s at 56°C, 20 s at 72°C. 18s rRNA was used for normalization.

## Results

### Sequencing of China Antique lotus

Illumina single-end sequencing technology was used to generate digital expression signatures for China Antique lotus under heat shock stress. Using RNA-seq technology, we sequenced four libraries, specifically, two biological replicates for control plant samples under 23°C (C1,C2) and two for heat stress samples treated for 1 h at 37°C after transfer (H1, H2). In total, the four libraries generated between 7.1 and 9.2 million raw reads. After removing reads containing adapters or poly-N and low quality reads, the total number of clean reads per library ranged from 7.1 to 9.1 million. Simultaneously, Q20 and GC contents of clean data were calculated. The Q20 values were >98% and GC content was relatively stable at ~46%, indicating the high quality of the sequencing libraries (Table 1).

**Table 1:** Sequence statistics of *Nelumbo nucifera* under heat stress.

Sample name	Rawreads	Clean reads	clean bases	Error rate(%)	Q20(%)	Q30(%)	GC content(%)
C1	9,209,534	9,134,374	0.46G	0.01	98.78	96.05	46.2
C2	9,078,297	8,952,858	0.45G	0.01	98.77	96.03	46.19
H1	7,133,967	7,067,033	0.35G	0.01	98.75	95.91	46.95
H2	8,430,780	8,355,964	0.42G	0.01	98.79	96.07	46.29

Q20, Q30: 20, 30 are Phred scores; Qphred = -10log10(e); Q20 and Q30 represent sequencing error rates of 0.01 and 0.001, respectively.

### Mapping sequences to the *Nelumbo nucifera* reference genome

To reveal the molecular events associated with RNA-seq data, single-end clean reads were aligned to the *Nelumbo nucifera* reference genome (http://www.ncbi.nlm.nih.gov/genome/genomes/14095) using TopHat. Compared with the non-splice mapping tool, TopHat usually obtains better results for the generation of a splice junction database based on gene model annotation. Consequently, 6,528,866 to 8,771,183 clean reads were mapped to the reference genome (Table 2), accounting for 92% to 96% of the total clean reads. Only 4–8% of total reads could not be mapped to the *Nelumbo nucifera* genome. These tags may represent regions where the references are incomplete.

**Table 2:** Summary of statistics for mapping reads to the *Nelumbo nucifera* genome.

Sample name	C1	C2	H1	H2
Total reads	9,134,374	8,952,858	7,067,033	8,355,964
Total mapped	8,771,183 (96.02%)	8,554,955 (95.56%)	6,528,866 (92.38%)	8,022,425 (96.01%)
Multiple mapped	251,965 (2.76%)	234,005 (2.61%)	193,682 (2.74%)	238,838 (2.86%)
Uniquely mapped	8,519,218 (93.27%)	8,320,950 (92.94%)	6,335,184 (89.64%)	7,783,587 (93.15%)
Reads map to '+'	4,258,216 (46.62%)	4,158,782 (46.45%)	3,163,685 (44.77%)	3,891,755 (46.57%)
Reads map to '-'	4,261,002 (46.65%)	4,162,168 (46.49%)	3,171,499 (44.88%)	3,891,832 (46.58%)
Non-splice reads	7,325,801 (80.2%)	7,151,871 (79.88%)	5,479,746 (77.54%)	6,738,944 (80.65%)
Splice reads	1,193,417 (13.07%)	1,169,079 (13.06%)	855,438(12.1%)	1,044,643 (12.5%)

Among the mapped reads, mapping ratios to exon, intron and intergenic regions were calculated. Over 94% reads could be mapped to the exon regions. Reads mapped to the intron regions (2.30% to 2.5%) were most likely derived from residue of pre-mRNA or stranded introns in the alternative splicing process. Approximately 3% reads were mapped to intergenic regions, probably owing to incomplete annotation of the genome (Fig A in S1 File).

The gene expression levels were estimated based on RPKM values [13]. The normalized final counts are summarized in Table B in S1 File, and RPKM density distribution in Fig B in S1 File. Only a small number of genes displayed relatively high expression, and over 75% genes had fewer than 15 reads. To evaluate the quality of RNA-seq data, analysis of the mean coverage of gene distribution was performed. As shown in Fig C in S1 File, all the libraries showed bell-shaped distribution from 5’ to 3’ and were highly normalized. In addition, relatively high correlation was observed between biological replicates, with Pearson correlation values of 0.96 and 0.94 for control and heat stress treatments, respectively (Fig D in S1 File).

### Analysis of differential gene expression

**Fig 1:**
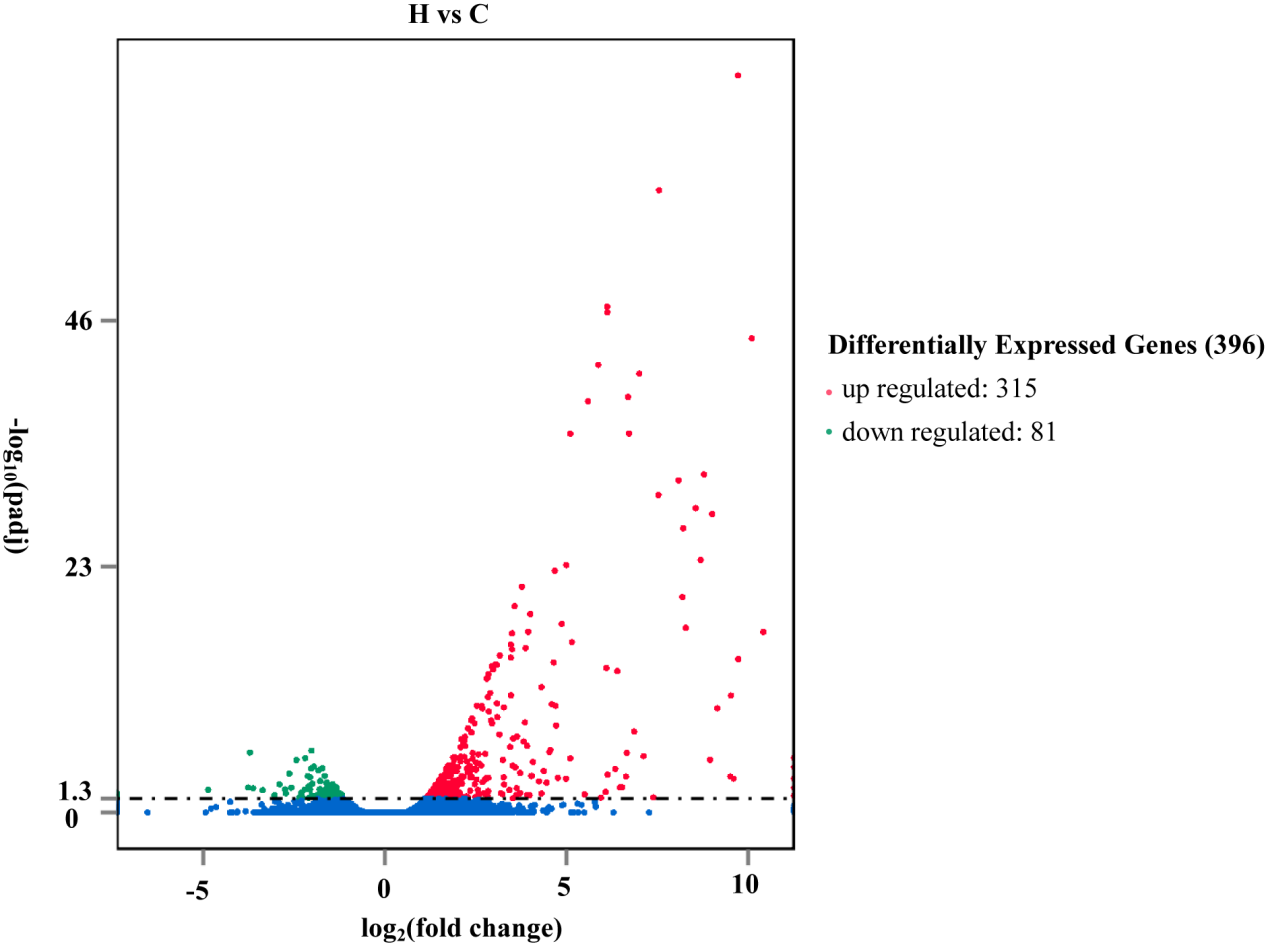
Volcano plot of differentially expressed genes under heat stress in *Nelumbo nucifera*. To eliminate biological variation of DESeq, screening criterion for differentially expressed genes is Padj <0.05. Log2 Ratio, log fold changes using the ratio base 2 logarithm.

To compare gene expression profiles of heat shock-exposed and control plants, DEseq was applied for differential gene expression analysis [14]. Genes with an adjusted *P*-value <0.05 were categorized as differentially expressed. A total of 396 genes with significant alterations were detected across the genome, among which 315 were upregulated while 81 were downregulated by heat shock stress (Fig 1). To evaluate the genome-wide expression profiles between control and heat-treated plants, we performed linkage hierarchical clustering using comparative data (Fig E in S1 File). The cluster indicated that some gene transcripts were in high abundance under both heat treatment and control conditions, while others display specific expression changes by heat stress.

### Gene Ontology enrichment

**Fig 2:**
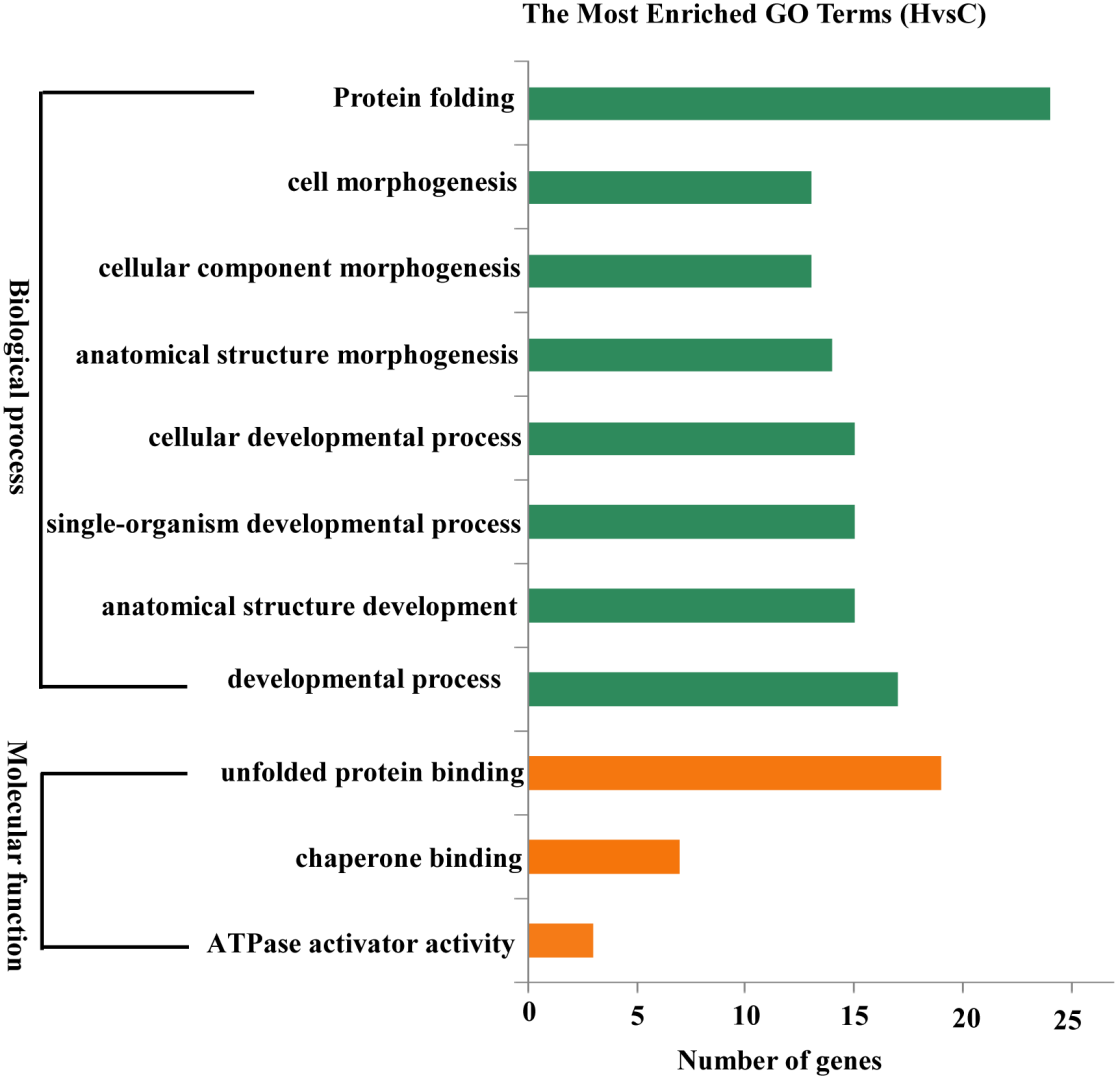
Histogram of Gene Ontology (GO) classification using GOseq. GO terms with corrected P-values <0.05 were considered significantly enriched for differentially expressed genes.

To functionally classify the genes affected by heat shock stress, Gene Ontology (GO) enrichment of differentially expressed genes were investigated. Based on Wallenius non-central hyper-geometric distribution, genes were categorized using GOseq software [15]. As a result, 296 (74.74%) differentially expressed genes were annotated, and 11 specific terms were significantly enriched (corrected *P*-value < 0.05) in two main categories: biological process and molecular function. Notably, protein folding (GO:0006457), cell morphogenesis (GO:0000902) and cellular component morphogenesis (GO:0032989) were identified as the top three terms under heat shock stress (Fig 2, Table C in S1 File). TopGO analysis indicated that protein folding and cell morphogenesis were the most important biological processes (Fig F in S1 File), while specific enrichment of unfolded protein binding and chaperone binding were observed for molecular function (Fig G in S1 File).

### Metabolic pathway analysis of differentially expressed genes using KEGG

**Fig 3:**
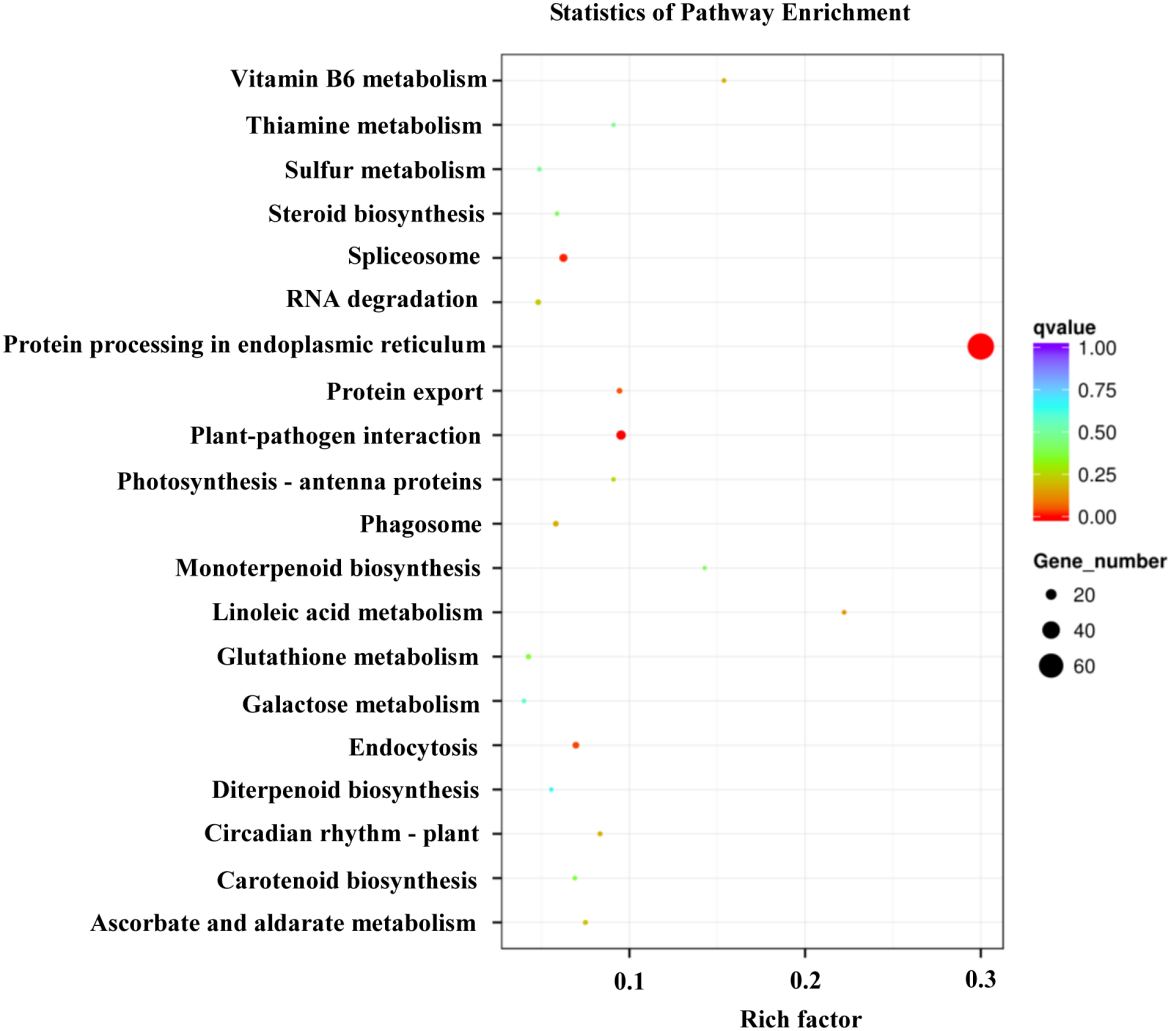
Scatter plot showing enrichment of differentially expressed genes (DEG) with KEGG classification. Pathways with q values ≤0.05 were significantly enriched in DEGs.

To further determine the biological functions of differentially expressed genes, we mapped these genes to terms in the KEGG database. Among the mapped pathways, four were significantly enriched (Corrected *P*-value≤0.05) under heat shock stress. Notably, protein processing in endoplasmic reticulum, plant-pathogen interaction, spliceosome, endocytosis, and protein export pathways were specifically enriched (Fig 3). Detailed information on the protein processing in endoplasmic reticulum pathway in the KEGG database indicated that ER-associated degradation and ubiquitin ligase complex are markedly affected by heat stress (Fig H in S1 File).

### Heat-shock proteins and molecular chaperones in China Antique lotus

In plants, Hsps/chaperones responsible for the events of protein folding, assembly, translocation and degradation play a crucial role in heat stress [7]. Data from this study revealed upregulation of heat-shock proteins and molecular chaperones under conditions of heat stress in lotus. As shown in Table 3, a total of 74 Hsps/chaperones were identified, including Hsp100 (Clp), Hsp90, Hsp70, chaperones (Hsp60) and small Hsp (sHsp) family members. Among these, 27 genes belonged to the sHsp family, accounting for 36.49% of the Hsps/chaperones identified. In terms of gene expression levels, 57.69% Hsps/chaperones were more than 3-fold increased under heat stress. Remarkably, 13 sHsps (nearly 50%) showed greater than 7-fold increased expression, indicating an important role in protein processing in lotus (Table 3).

**Table 3:** HSPs and molecular chaperones upregulated by heat stress in *Nelumbo nucifera*.

Gene_id	log2FoldChange	padj	Description
LOC104611040	6.12	1.71E-47	Hsp100/chaperone protein ClpB1
LOC104598003	2.04	3.75E-02	Hsp100/chaperone protein ClpB3, chloroplastic
LOC104594214	1.65	3.49E-04	heat shock protein 90–1
LOC104597578	2.85	5.74E-04	activator of 90 kDa heat shock protein ATPase
LOC104597584	3.09	1.54E-14	activator of 90 kDa heat shock protein ATPase
LOC104597851	1.63	4.03E-03	heat shock protein 81–1
LOC104600195	6.12	4.87E-48	heat shock protein 83
LOC104600243	1.29	1.17E-02	heat shock protein 83
LOC104606598	3.09	1.23E-09	heat shock protein 81–1
LOC104611516	6.69	1.38E-39	heat shock protein 83
LOC104586952	1.38	5.16E-03	stromal 70 kDa heat shock-related protein
LOC104588447	2.38	3.29E-08	heat shock 70 kDa protein, mitochondrial
LOC104590620	2.16	3.98E-07	heat shock 70 kDa protein 17
LOC104592243	1.75	3.94E-05	heat shock 70 kDa protein 15
LOC104594564	3.04	1.40E-14	heat shock cognate 70 kDa protein 2
LOC104594575	2.11	1.39E-07	heat shock cognate 70 kDa protein 2
LOC104595227	1.69	1.16E-04	heat shock 70 kDa protein 15
LOC104596515	2.52	2.25E-05	heat shock 70 kDa protein 8
LOC104609133	9.01	1.22E-28	heat shock 70 kDa protein
LOC104609134	10.4	1.29E-17	heat shock 70 kDa protein
LOC104609993	2.00	5.65E-05	heat shock 70 kDa protein 17
LOC104603244	10.10	4.59E-45	heat shock 70 kDa protein
LOC104601069	3.47	3.30E-15	hsp70-binding protein 1
LOC104602273	1.77	4.37E-05	hsp70-Hsp90 organizing protein 3
LOC104598594	3.53	1.21E-07	hsp70-Hsp90 organizing protein 3
LOC104604657	6.54	4.56E-03	25.3 kDa heat shock protein, chloroplastic
LOC104585845	8.79	2.44E-32	18.2 kDa class I heat shock protein
LOC104590850	9.73	4.67E-15	17.8 kDa class I heat shock protein
LOC104592442	3.43	8.00 E-03	26.5 kDa heat shock protein, mitochondrial
LOC104593906	4.65	9.29E-15	17.6 kDa class I heat shock protein
LOC104599794	9.61	6.87E-04	17.8 kDa class I heat shock protein
LOC104600474	6.40	6.02E-14	17.4 kDa class III heat shock protein
LOC104600496	2.58	2.54E-05	20 kDa chaperonin, chloroplastic
LOC104600625	6.34	8.51E-05	17.1 kDa class II heat shock protein
LOC104601155	5.11	3.78E-36	17.3 kDa class I heat shock protein
LOC104607385	2.57	3.71E-06	15.7 kDa heat shock protein, peroxisomal
LOC104607800	4.25	1.23E-03	17.8 kDa class I heat shock protein
LOC104607801	9.15	1.81E-10	17.8 kDa class I heat shock protein
LOC104607802	8.95	1.20E-05	17.5 kDa class I heat shock protein
LOC104607836	8.69	2.42E-24	17.8 kDa class I heat shock protein
LOC104607837	9.72	1.14E-69	17.8 kDa class I heat shock protein
LOC104607838	7.39	3.93E-02	17.8 kDa class I heat shock protein
LOC104607839	8.56	3.46E-29	17.8 kDa class I heat shock protein
LOC104607840	9.53	1.18E-11	17.8 kDa class I heat shock protein
LOC104608468	9.51	4.46 E-04	17.1 kDa class II heat shock protein
LOC104609165	5.95	4.07E-02	17.3 kDa class I heat shock protein
LOC104609166	6.47	4.52E-03	17.3 kDa class I heat shock protein
LOC104609367	8.28	5.46E-18	17.1 kDa class II heat shock protein
LOC104604658	3.28	2.44 E-03	17.8 kDa class I heat shock protein
LOC104592981	4.99	7.08E-04	small heat shock protein, chloroplastic
LOC104598537	6.86	2.74E-08	small heat shock protein, chloroplastic
LOC104602326	8.19	7.25E-21	small heat shock protein, chloroplastic
LOC104602123	1.88	7.48E-06	chaperonin CPN60-2, mitochondrial
LOC104587652	2.38	2.38E-09	chaperonin CPN60-2, mitochondrial
LOC104600478	1.93	7.68E-06	chaperone protein dnaJ 6
LOC104608050	2.67	1.12E-10	dnaJ homolog subfamily B member 1
LOC104609916	4.45	1.63E-03	dnaJ homolog subfamily B member 8
LOC104585999	1.86	1.48E-04	chaperone protein dnaJ 1, mitochondrial
LOC104589095	3.17	2.15E-15	dnaJ homolog subfamily B member 13
LOC104594942	2.47	2.21E-03	dnaJ protein ERDJ3A
LOC104605116	3.08	6.29E-11	dnaJ protein ERDJ3B
LOC104602647	2.05	8.17E-06	dnaJ protein P58IPK homolog
LOC104598847	2.52	8.24E-04	dnaJ homolog subfamily B member 3
LOC104599473	2.21	6.02E-07	dnaJ homolog subfamily B member 1
LOC104605136	4.04	3.81E-04	dnaJ homolog subfamily B member 3
LOC104601430	1.50	9.22E-04	dnaJ protein
LOC104601432	2.81	3.14E-13	dnaJ protein
LOC104606412	1.60	5.21E-04	20 kDa chaperonin, chloroplastic
LOC104600655	3.77	7.96E-22	10 kDa chaperonin
LOC104600279	6.64	4.41E-04	BAG family molecular chaperone regulator 6
LOC104594149	2.34	2.22 E-04	BAG family molecular chaperone regulator 5, mitochondrial
LOC104595069	4.87	2.27E-18	T-complex protein 1 subunit gamma
LOC104591565	1.44	2.44 E-03	T-complex protein 1 subunit epsilon
LOC104601564	1.23	3.02 E-02	T-complex protein 1 subunit zeta

Screening criteria for upregulated genes (DEGs): *P*adj <0.05. Log2 Ratio, log fold changes using the ratio base 2 logarithm.

### Cell morphogenesis-related genes in China Antique lotus

We identified 19 genes related to cell morphogenesis and cellular component morphogenesis that were significantly induced by heat stress, including cell wall structural genes, xyloglucan related genes, transmembrane genes, aquaporins, extensins and lipid transfer genes. Four xyloglucan-related genes (LOC104591922, LOC104599119, LOC104607928, LOC104591734) were identified as heat-inducible, supporting a critical role of cell wall remodeling under conditions of heat stress in *Nelumbo nucifera*. Increased expression of three aquaporins (LOC104603647, LOC104594630, LOC104604560) was additionally observed, indicating positive roles during the early stages of heat stress in lotus (Table 4).

**Table 4:** Proteins related to cellular morphogenesis upregulated by heat stress in *Nelumbo nucifera*.

Gene_id	log2FoldChange	padj	Description	Subcellular prediction
LOC104587372	2.05	1.45E-02	glycine-rich cell wall structural protein	-
LOC104610879	2.82	5.88E-04	transmembrane protein	Vacuole
LOC104603094	1.74	8.42E-05	transmembrane protein	Nucleus
LOC104605640	2.49	3.68E-02	secretory carrier-associated membrane protein	Plasma membrane
LOC104608147	2.58	1.98E-02	extensin-2	Nucleus
LOC104608452	3.73	1.99E-04	extensin-2	Secreted
LOC104602657	1.80	1.83E-02	tetraspanin-6	Plasma membrane
LOC104607928	1.30	2.62E-02	protein altered xyloglucan 4	Cytoplasm, Secreted
LOC104591922	1.80	1.03E-04	xyloglucan endotransglucosylase/hydrolase protein	Secreted
LOC104599119	3.59	6.13E-05	xyloglucan endotransglucosylase/hydrolase protein	Secreted
LOC104591734	1.79	4.36E-02	glucuronoxylan 4-O-methyltransferase	Nucleus
LOC104593176	1.74	1.92E-03	Polygalacturonase	Secreted
LOC104594630	1.50	1.13E-03	aquaporin TIP1-3	Chloroplast
LOC104603647	1.56	4.23E-04	aquaporin PIP2-7	Plasma membrane
LOC104604560	1.71	8.43E-05	aquaporin TIP1-1	Cytoplasm, Membrane
LOC104597663	4.31	1.93E-12	lipid-transfer protein	Secreted
LOC104607503	1.39	7.23E-03	1-acylglycerol-3-phosphate O-acyltransferase	Nucleus

Screening criterion for upregulated genes (DEGs): *P*adj <0.05. Log2 Ratio, log fold changes using the ratio base 2 logarithm. The subcellular locations were predicted from The Plant Secretome and Subcellular Proteome KnowledgeBase (http://bioinformatics.ysu.edu/secretomes/plant/index.php).

### Quantitative real-time PCR data

**Fig 4:**
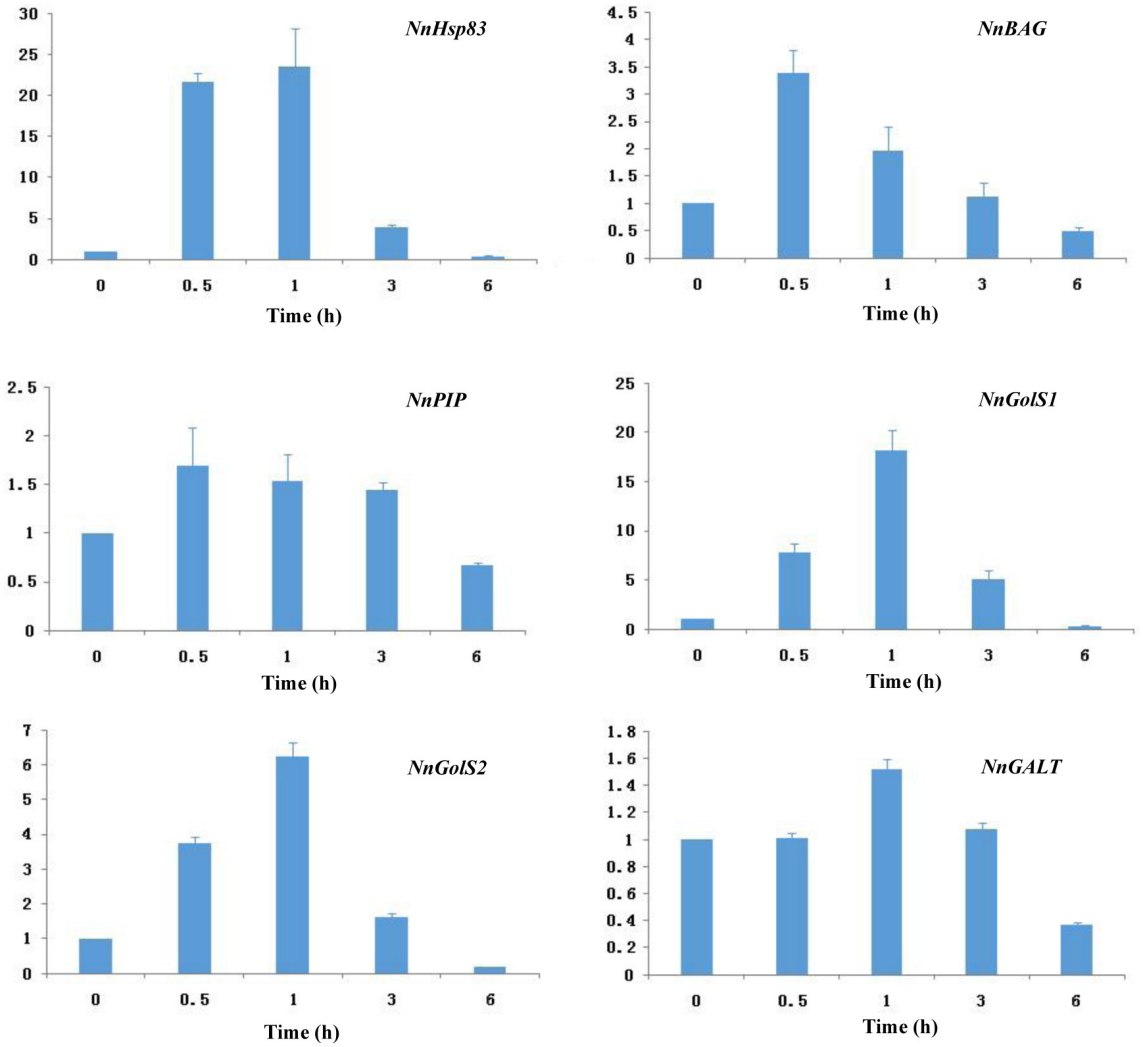
Analysis of genes upregulated by heat stress in *Nelumbo nucifera* using quantitative RT-PCR. 18s rRNA was used for normalization.

To validate RNA-seq data, expression patterns of candidate genes were further analyzed using quantitative qRT-PCR. As shown in Fig 4, *NnHsp83*, *NnBAG*, *NnPIP*, *NnGolSs*, *NnGALT* were upregulated by heat stress, but the induction patterns varied among those genes. Generally, the findings were consistent with RNA-seq results. As verified by qRT-PCR, two *NnGolS* genes were significantly induced by heat stress, with an expression peak at 1 h, indicating a fast-acting role of raffinose biosynthesis under heat stress (Fig 4).

## Discussion

### Analysis of differentially expressed genes

Response to heat stress is a complex phenomenon involving extensive gene expression changes in plants [7]. Under heat shock stress, the number of induced genes is 3–6 times more than the number of repressed genes in maize, barley, wheat and rice [16–19]. Following long-term heat stress over 6 h, the proportions of heat-induced and suppressed genes were similar, with the number of upregulated genes being slightly lower than that of downregulated genes [17,20–21]. In this study, we focused on the response of lotus to heat shock stress. Overall, 396 genes were differentially expressed under heat stress conditions in lotus, with a 3.89 times greater number of upregulated than downregulated genes. The results indicate that the rapid response of lotus to heat shock stress shares similar characteristics with that of specific land plants. However, ~23.74% of the differentially expressed genes remained uncharacterized, since no homologs have been identified in the NCBI database. Some of these genes may represent novel heat-responsive transcripts unique to aquatic plants.

### Roles of Hsps and chaperones in heat response

Hsps/chaperones are conservatively divided into five major families: Hsp100 (Clp), Hsp90, Hsp70 (DnaK), chaperones (GroEL and Hsp60) and small Hsp (sHsp) [7,22]. These proteins play a crucial role in maintaining cellular homeostasis and functional conformation, and some Hsps/chaperones are correlated with acquisition of thermotolerance in *Arabidopsis* [16,22]. In this study, 74 Hsps/chaperones were identified in China Antique lotus, among which sHsps constituted the largest subfamily induced by heat stress.

sHsps are low molecular mass Hsps of 12–40 kDa [22]. Among the five families of Hsps, sHsps are the most prevalent in terms of cellular location with diverse functions in plants [23–24]. sHsps generally function as molecular chaperones through binding, stabilizing and preventing non-native aggregation, facilitating subsequent refolding [25–27]. In *Arabidopsis*, around 13 sHsps have been identified [22], with some playing a non-redundant role in acquired thermotolerance [28]. In tobacco, chloroplast-localized sHsp protects photosystem II to ensure survival under heat stress conditions [29], and overexpression of mitochondrial sHsp has been shown to significantly enhance thermotolerance in tobacco [30]. Wang and co-workers (2004) revealed that accumulation of sHsp is strongly correlated with plant thermotolerance in *Arabidopsis*. In China Antique lotus, 27 sHsps were significantly elevated by heat stress, representing twice the number of sHsps, relative to *Arabidopsis*. In terms of cellular location, sHsps accumulating under heat stress conditions in lotus were predicted as chloroplastic, mitochondrial and peroxisomal. The abundance and diversity of sHsps in lotus suggests an important role in acquired thermotolerance.

Hsp70 chaperones, together with co-chaperones (e.g., DnaJ/Hsp40), assist in a range of protein folding processes [22]. In *Arabidopsis*, at least 18 genes encoding members of the Hsp70 family have been identified. Some members are significantly induced by heat stress, as observed using expression profile analysis [31–32]. Hsp70 interacts directly with DnaJ/Hsp40 cochaperones and unfolded proteins to regulate interactions, thereby correcting protein folding in *Arabidopsis* [33–35]. In this study, 12 genes encoding Hsp70 and 12 encoding DnaJ members were significantly induced by heat stress. In view of these results, it is reasonable to suggest that Hsp70 interacts with DnaJ/Hsp40 cochaperones to facilitate correct protein folding under heat stress in lotus, potentially playing a positive role in acquisition of thermotolerance.

Recent studies have shown that HSP70 proteins interact directly with Bcl-2-associated athanogene (BAG) proteins and modulate their activity under heat stress [36]. Seven members of the *Arabidopsis* BAG protein family have been identified to date. Among these, AtBAG6 is significantly induced by heat stress and AtBAG7 interacts directly with AtBiP2 proteins to regulate their activity under heat stress [36]. In lotus, two BAG family genes were identified as heat-inducible genes, and one mitochondrial BAG gene was predicted to be a homolog of *AtBAG5*, an ancient BAG gene in plants. However, the interactions between the BAG family and Hsp70 in lotus require further experimental research.

### Expression analysis of genes related to cell morphogenesis

In general, genes related to cell growth, expansion and cell wall biosynthesis are repressed by heat stress, signifying a negative effect of heat stress on cell physiology in plants. In *Arabidopsis* shoots and barley caryopses, downregulation of genes related to extensins, cell membrane, lipid binding and dehydrin is commonly observed as a result of cellular damage under heat stress [17,37]. In contrast, genes related to cell morphogenesis were rapidly induced under heat stress after only 1 h in China Antique lotus. Examples of elevated genes included extensins, cell wall structural genes, xyloglucan-related genes and lipid transfer genes (Table 4). Upregulation of these genes may be critical for cell remodeling, growth and expansion under heat stress. Moreover, levels of two *TIPs* and one *PIP* were upregulated in lotus, indicating an active role in rapid water uptake and transportation under heat stress. Positive expression of aquaporin genes have been reported in rice, with constitutive upregulation of *OsNIP2;1* and *OsTIP1;2* genes during heat stress [19,38]. The rapid induction of genes related to cell morphogenesis in China Antique lotus may facilitate re-establishing of the cellular balance to cope with heat stress. Based on the collective findings, we propose that stabilization and maintenance of cell morphogenesis and physiology contribute to acquired thermotolerance in lotus.

## Conclusions

In this study, we characterized the digital gene expression signatures for China Antique lotus under heat shock stress conditions, leading to the identification of significantly enriched pathways and a number of candidate genes involved in heat stress response. Data from this study provide valuable clues that may aid in understanding the molecular events underlying response to heat stress in China Antique lotus.

## Supporting Information

S1 File Supporting tables and ures. (PDF)
