# Supplementary material for: Gene Expression Profile in the Long-Living Lotus: Insights into the Heat Stress Response Mechanism
Source: PLoS One. 2016 Mar 28;11(3):e0152540. doi: 10.1371/journal.pone.0152540 (PMC4809550; doi:10.1371/journal.pone.0152540)
Supplement: S1 File — (PDF) [file pone.0152540.s001.pdf]

Table A The gene list and primer pairs used in quantitative Real-Time PCR analysis.

Gene id	Gene	Forward primer	reverse primer
LOC104611516	Hsp83	5-CCCTGCTGTTTGGTTACTGGAG-3	5-CCTTCACCGATTTGTCATTCTTG-3
LOC104594149	BAG	5-CCTCCCTCAAAGCACGATTCTC-3	5-TCGTCAGGAGGCGCTTGAGTTT-3
LOC104603647	PIP	5-TTATCGGTCACTCAAAGCAGGG-3	5-CTGAGTTAGCACCACCTCCATTG-3
LOC104607072	Gols	5-TAAGCCCATTCCTCTGATCTACAAC-3	5-CACATCCCACCATTTCTTCACA-3
LOC104611225	Gols	5-TACGCAGTGATGGATTGTTTCTG-3	5-CAAGAGATCGTCGCAGGTAGAG-3
LOC104612986	GALT	5-CAACATCGTCCTGACTCCCG-3	5-GGAACATCCTTACCGCACATC-3
M82398.1	18S rRNA	5-TAGGATAGTGGCCTACTATGGT-3	5-TTAAGGGATTTAGATTGTACTCA-3

Table B Statistics of gene abundance.

**RPKM Interval**	**C1**	**C2**	**H1**	**H2**
0~1	9673(34.47%)	9286(33.09%)	9718(34.63%)	9674(34.48%)
1~3	4113(14.66%)	4356(15.52%)	4449(15.86%)	4265(15.20%)
3~15	7899(28.15%)	8052(28.70%)	7894(28.13%)	8116(28.92%)
15~60	4536(16.17%)	4533(16.16%)	4241(15.11%)	4297(15.31%)
>60	1838(6.55%)	1832(6.53%)	1757(6.26%)	1707(6.08%)

RPKM： expected number of reads Per Kilobase of transcript sequence per Millions base pairs sequenced.

Table C GO enrichment of differently expressed genes.

GO_accession	Description	Term_type	Corrected_pValue
GO:0006457	protein folding	biological_process	3.21E-11
GO:0000902	cell morphogenesis	biological_process	6.17E-09
GO:0032989	cellular component morphogenesis	biological_process	6.17E-09
GO:0009653	anatomical structure morphogenesis	biological_process	2.31E-06
GO:0048869	cellular developmental process	biological_process	4.61E-06
GO:0044767	single-organism developmental process	biological_process	3.31E-05
GO:0048856	anatomical structure development	biological_process	9.14E-05
GO:0032502	developmental process	biological_process	0.009104
GO:0051087	chaperone binding	molecular_function	0.004508
GO:0051082	unfolded protein binding	molecular_function	6.17E-09
GO:0001671	ATPase activator activity	molecular_function	0.039859

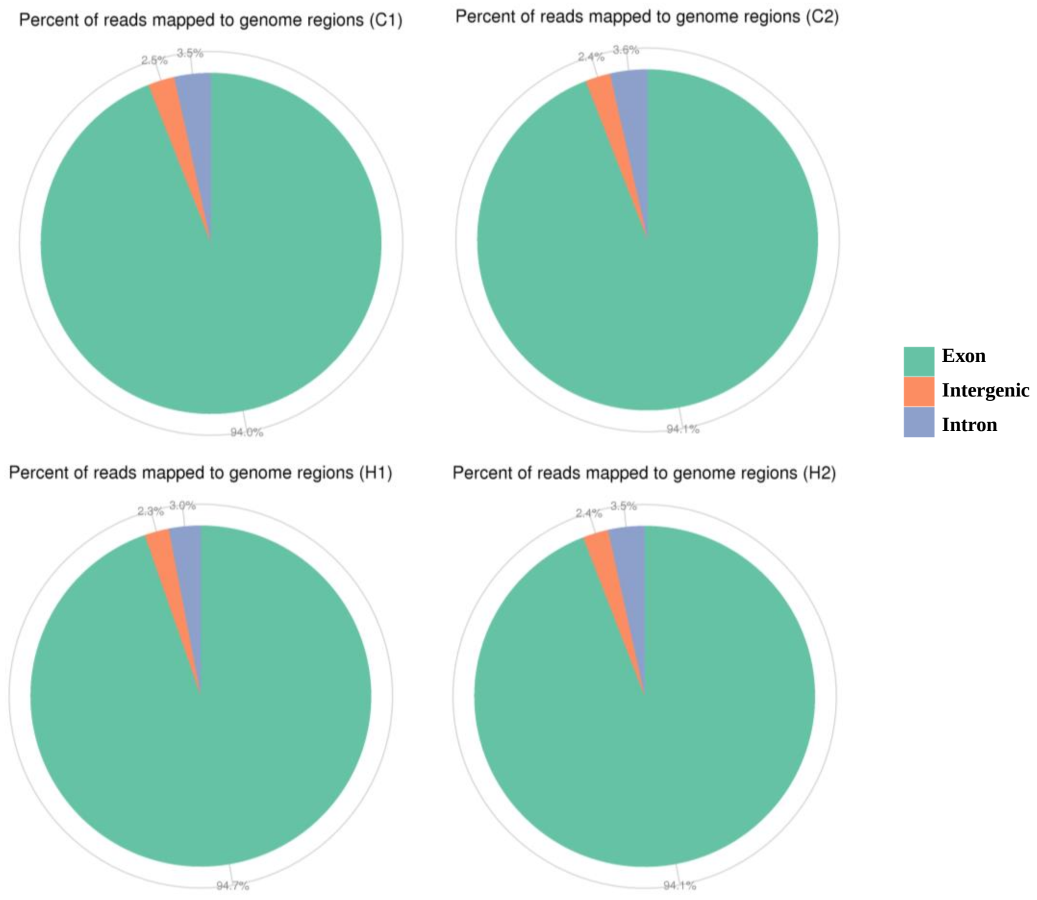

Figure A Mapping percentage to exon, intron and intergenic regions.

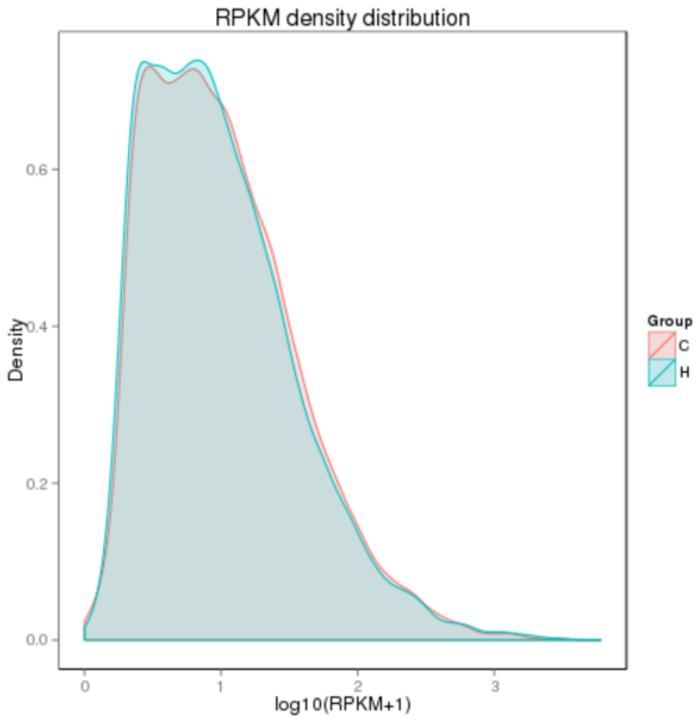

Figure B RPKM density distribution.

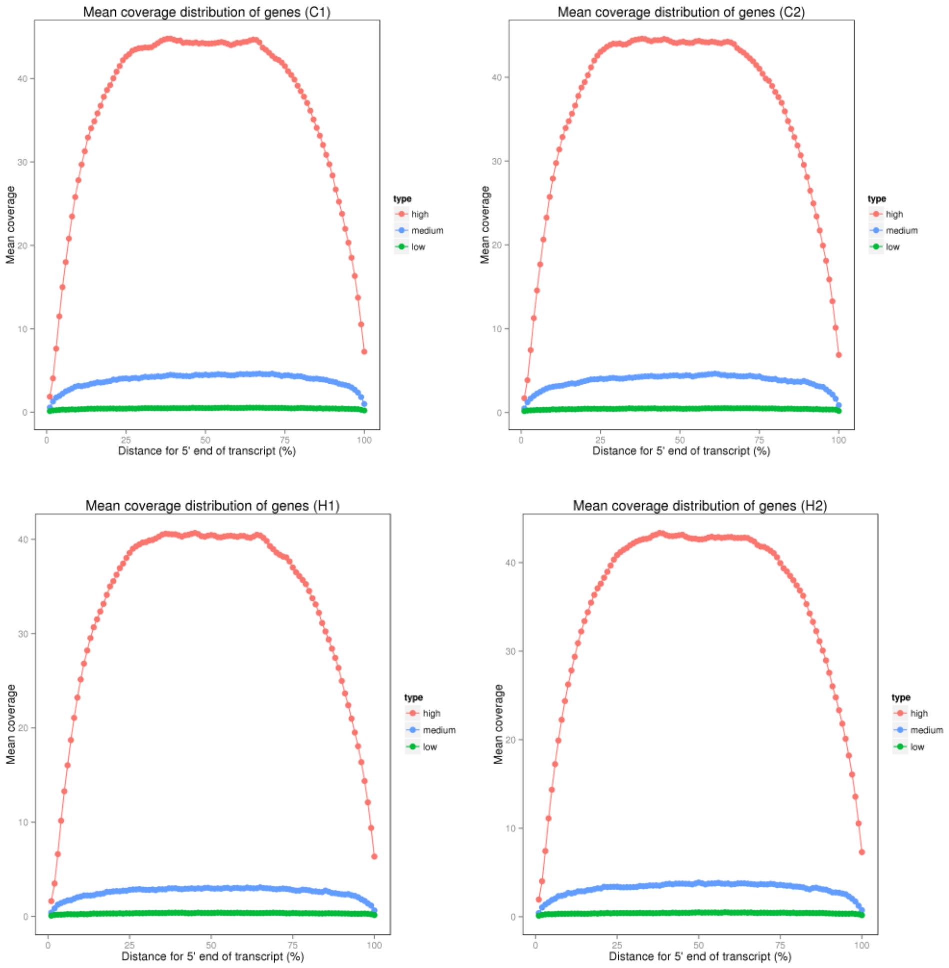

Figure C Mean coverage distribution of genes.

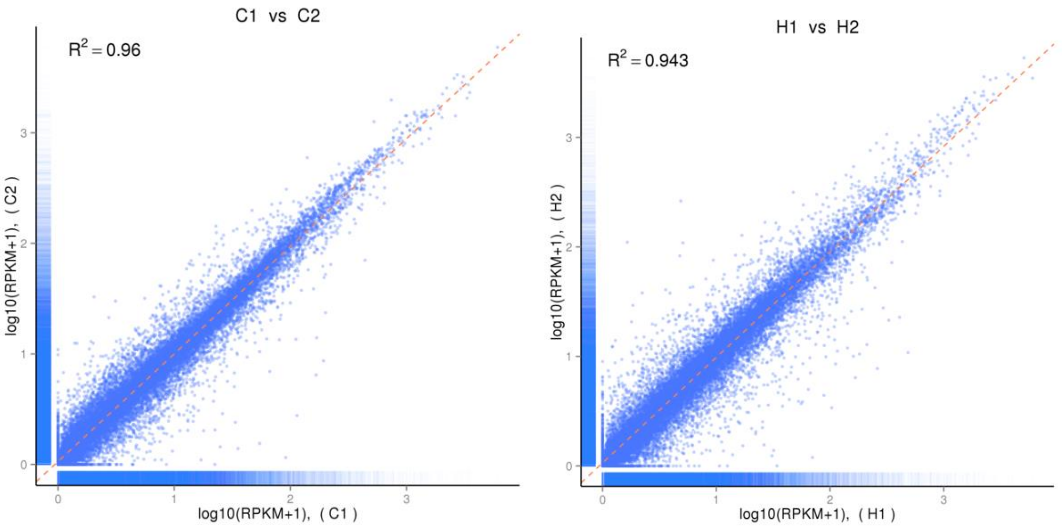

Figure D Pearson correlations of biological repeats.

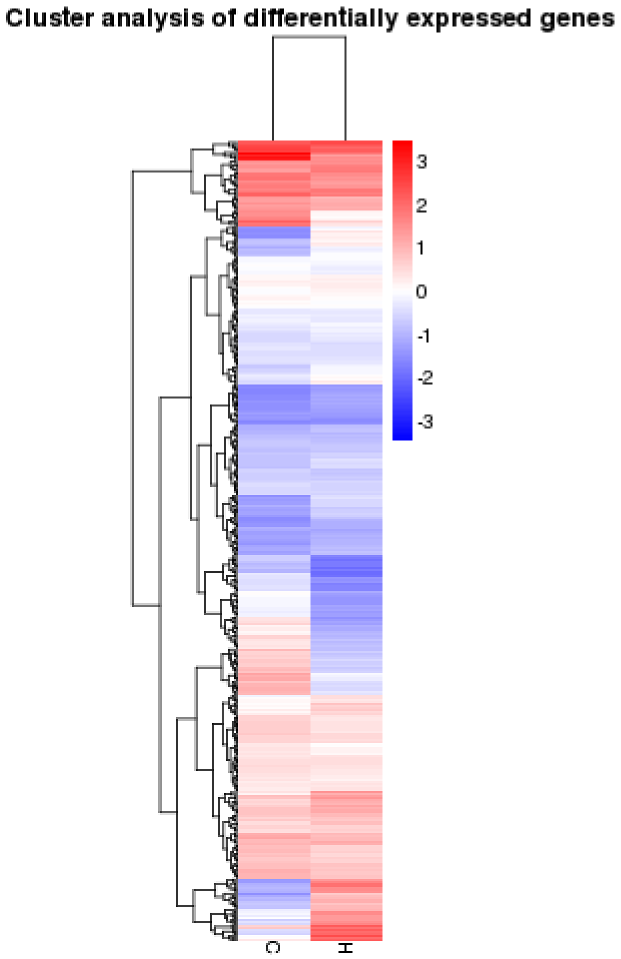

Figure E Hierarchical clustering of differentially expressed genes.

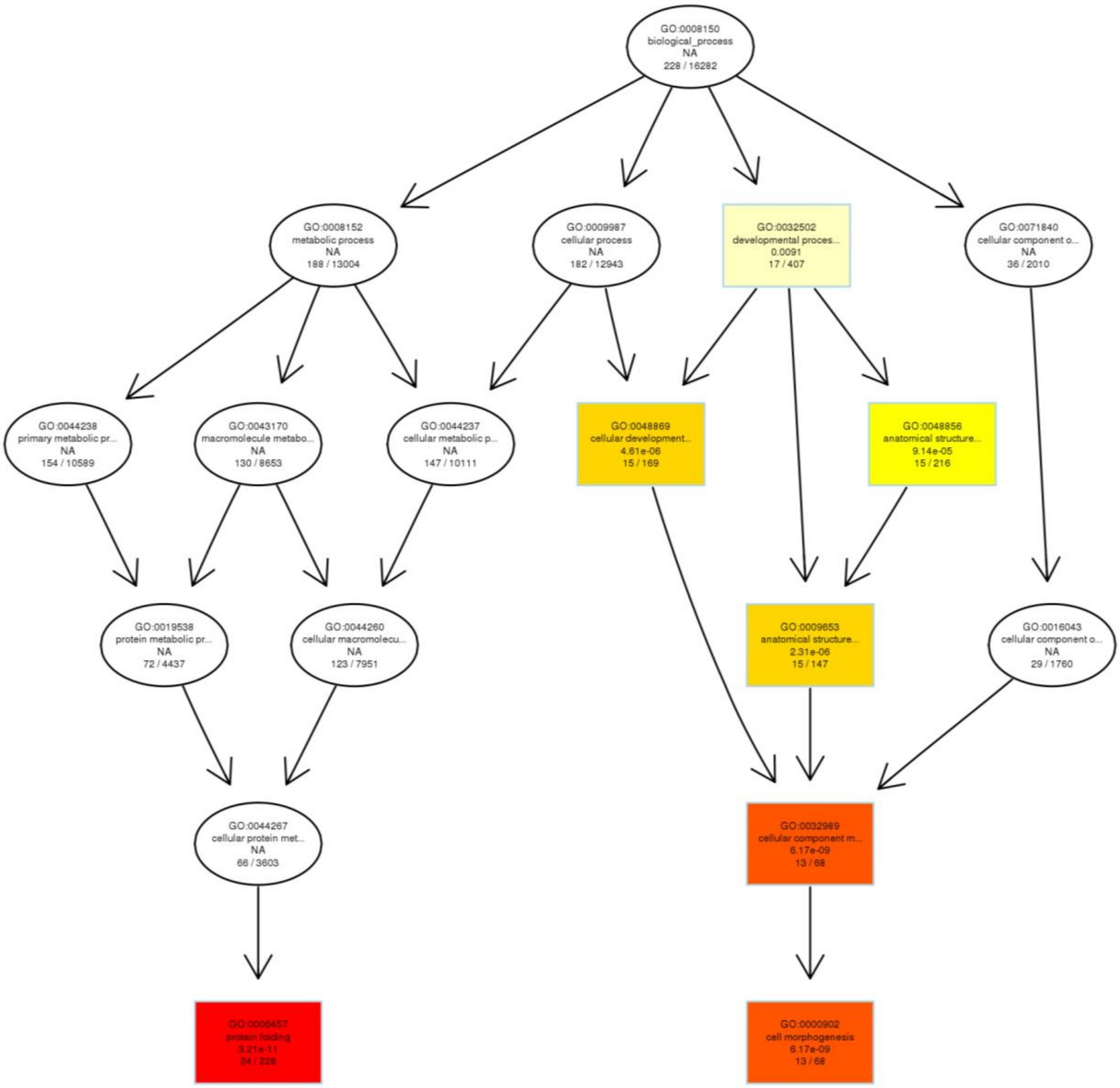

Figure F Directed Acyclic Graph of GO analysis concerning heat responsive genes in biological process using topGO. The color from white to red represents the degree of enrichment, the deeper the color, the higher degree of enrichment.

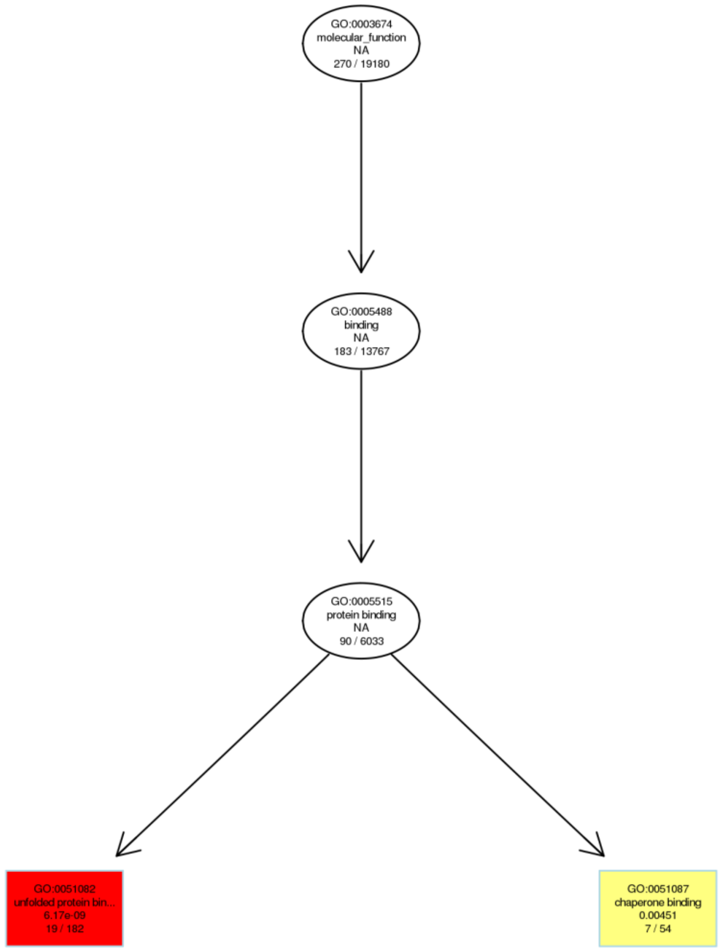

Figure G Directed Acyclic Graph of GO analysis concerning heat responsive genes in molecular function category using topGO. The color from white to red represents the degree of enrichment, the deeper the color, the higher degree of enrichment.

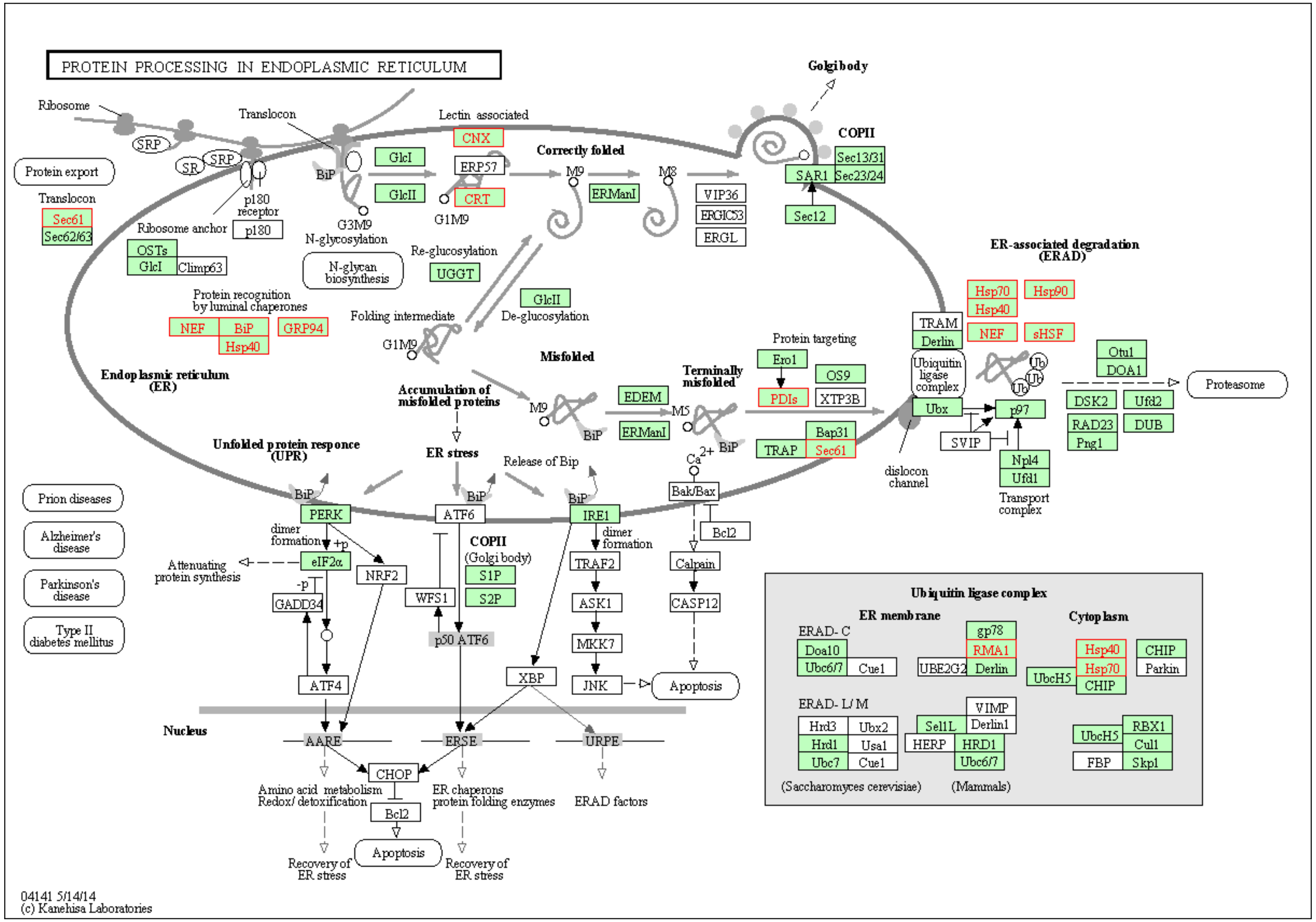

Figure H Detailed information of protein processing in endoplasmic reticulum pathway in KEGG database. In the figure, upregulated genes are marked with red color while down-regulated genes are marked with green color.
